# Deep learning system for distinguishing optic neuritis from non-arteritic anterior ischemic optic neuropathy at acute phase based on fundus photographs

**DOI:** 10.3389/fmed.2023.1188542

**Published:** 2023-06-29

**Authors:** Kaiqun Liu, Shaopeng Liu, Xiao Tan, Wangting Li, Ling Wang, Xinnan Li, Xiaoyu Xu, Yue Fu, Xiaoning Liu, Jiaming Hong, Haotian Lin, Hui Yang

**Affiliations:** ^1^State Key Laboratory of Ophthalmology, Zhongshan Ophthalmic Center, Sun Yat-sen University, Guangzhou, China; ^2^School of Computer Science, Guangdong Polytechnic Normal University, Guangzhou, China; ^3^Department of Ophthalmology, Shenzhen Aier Eye Hospital Affiliated to Jinan University, Shenzhen, Guangdong, China; ^4^Department of Ophthalmology, Shenzhen Eye Hospital, Shenzhen, Guangdong, China; ^5^Department of Ophthalmology, the First Hospital of Nanchang, The Third Affiliated Hospital of Nanchang University, Nanchang, China; ^6^School of Medical Information Engineering, Guangzhou University of Chinese Medicine, Guangzhou, China

**Keywords:** artificial intelligence, acute phase, optic neuritis, non-arteritic anterior ischemic optic neuropathy, color fundus photographs

## Abstract

**Purpose:**

To develop a deep learning system to differentiate demyelinating optic neuritis (ON) and non-arteritic anterior ischemic optic neuropathy (NAION) with overlapping clinical profiles at the acute phase.

**Methods:**

We developed a deep learning system (ONION) to distinguish ON from NAION at the acute phase. Color fundus photographs (CFPs) from 871 eyes of 547 patients were included, including 396 ON from 232 patients and 475 NAION from 315 patients. Efficientnet-B0 was used to train the model, and the performance was measured by calculating the sensitivity, specificity, and area under the receiver operating characteristic curve (AUC). Also, Cohen’s kappa coefficients were obtained to compare the system’s performance to that of different ophthalmologists.

**Results:**

In the validation data set, the ONION system distinguished between acute ON and NAION achieved the following mean performance: time-consuming (23 s), AUC 0.903 (95% CI 0.827–0.947), sensitivity 0.796 (95% CI 0.704–0.864), and specificity 0.865 (95% CI 0.783–0.920). Testing data set: time-consuming (17 s), AUC 0.902 (95% CI 0.832–0.944), sensitivity 0.814 (95% CI 0.732–0.875), and specificity 0.841 (95% CI 0.762–0.897). The performance (κ = 0.805) was comparable to that of a retinal expert (κ = 0.749) and was better than the other four ophthalmologists (κ = 0.309–0.609).

**Conclusion:**

The ONION system performed satisfactorily distinguishing ON from NAION at the acute phase. It might greatly benefit the challenging differentiation between ON and NAION.

## Introduction

Demyelinating optic neuritis (ON) and non-arteritic anterior ischemic optic neuropathy (NAION) are acute optic neuropathies with overlapping clinical profiles. While differentiation between ON and NAION can present challenges, not all cases are complex and there may be a certain rate of misdiagnosis in some clinical scenarios, especially in underdeveloped areas (1). Multiple examinations are required to facilitate the differential diagnosis in some patients. Despite this, a misdiagnosis rate of 59.8% has been reported, with 16% of the patients receiving excessive magnetic resonance imaging, 16% receiving needless lumbar puncture, and 11% receiving unnecessary intravenous steroids application (2). The pathological mechanisms of ON and NAION were distinct, requiring different treatment regimens (3). Moreover, diligent follow-up following treatment is necessary to finalize the diagnosis in a specific challenging situation. Early diagnosis may have a significant benefit on the visual prognosis. However, the prompt diagnosis was difficult due to the shortage of experienced neuro-ophthalmologists and advanced ophthalmic devices, resulting in irreversible vision loss (4, 5).

Artificial Intelligence (AI) based on fundus photography has shown incredible differential ability on retinal disease. It has been utilized to screen for diabetic retinopathy (6), age-related macular degeneration (7), and glaucoma (8). It could even distinguish between gender and an optic disc of the left or right eye (9, 10). Also, it was used to measure retinal nerve fiber layer thickness in ON and NAION (11). Fundus photography is a routine, cost-effective, non-invasive examination technique used to diagnose ON and NAION (1). However, previous studies have not reported using AI to distinguish ON and NAION based on fundus photographs.

Therefore, we describe the use of AI analysis to help differentiate between ON and NAION from the acute phase based solely on color fundus photographs (CFPs) and to generate guidelines for distinguishing these conditions when they overlap clinically. The ultimate goal is to develop an effective, convenient, and cost-effective AI-aided diagnostic technique for improving the differential diagnosis efficiency of ON and NAION.

## Materials and methods

The research adhered to the tenets of the Declaration of Helsinki and has obtained the Ethics Committee approval from Zhongshan Ophthalmic Center (ZOC) (2021KYPJ002).

### Data inclusion

A total of 871 eyes from 547 patients with ON and NAION were included, which were within 3 months of onset. And pediatric patients were not included in our study. When bilateral eyes were affected, photographs of bilateral eyes were included; if unilateral eye was affected, only the affected eye was included, while the unaffected eye was excluded; each CFP represented a unique eye. All CFPs with ON or NAION in the data set were obtained from the neuro-ophthalmological expert (HY), and were acquired using two retinal cameras (TRC-50DX, TOPCON YAMAGATA Co., Ltd., Japan, and FF 450plus, Carl Zeiss Meditec AG, Germany). The diagnosis of each CFP was established by a combination of detailed medical history, clinical examination, ophthalmic imaging, or neurological imaging, including visual acuity (VA, Snellen charts), VF (Humphrey Visual Field Analyzer, Carl Zeiss Meditec, Dublin, CA, USA), OCT (Carl Zeiss Meditec, Jena, Germany) or Spectral OCT (Heidelberg Engineering, Heidelberg, Germany), visual evoked potentials (VEP), MRI, autoimmune antibodies tests (cell-based assay to search for AQP4 antibody), and patients with 6-month follow-up records to clarify that these records were used to further confirm the diagnosis based on the patient’s disease progression and response to medication over a 6-month period. CFPs that were blurry, grossly out of focus, or did not fully display the optic disc were excluded.

### The deep learning system (ONION) development

The deep learning system distinguishing ON from NAION at the acute phase using original CFPs, named ONION, was developed. We trained the ONION using one of the state-of-the-art convolutional neural network (CNN) algorithms, Efficientnet-B0. The Efficientnet-B0 algorithm was a fine-designed deep CNN model that Google proposed in recent years. Firstly, the training data set was utilized in the ONION system to optimize its parameters and produce candidate models. During the model training process, the batch size was set to 16, the focus loss was set to 0.5, and the number of training iterations was set to 50. Secondly, we performed a 10-fold cross-validation test (training 60%; validation 20%; and testing 20%) using CFPs from our data set for internal validation and model development. After the 10-fold cross-validation was completed, we then tested the cross-validation model with the best performance against the testing data set.

### Performance comparison between the ONION system and ophthalmologists

All study CFPs in the testing data set were evaluated independently by five trained graders [three fellowships with 2, 3, and 5 years of fellowships (LW, XT, and YF), one board certified ophthalmologists with over 10 years of clinical experience (XLiu) and one retinal expert (XX)] who were masked to the diagnosis in either eye. These graders were classified independently based on CFPs alone, and these CFPs were classified to ON or NAION. CFPs grading was performed at a centralized reading center using high-resolution, high-definition LCD computer displays. These display monitors were regularly color-calibrated to a color temperature of 6,500 K and gamma setting of 2.2 (Spyder4PRO; Datacolor, Lawrenceville, NJ, USA).

### Visualization of photographs features

We created heatmaps through class activation mapping (CAM) to identify the key regions in the CFPs used by the EfficientNet to classified ON and NAION. Briefly, the CAM was obtained by projecting back the weights of the EfficientNet model’s output layer onto the convolutional feature, which could identify the class-specific discriminative regions, namely, the specific regions in the CFPs with the highest impact on the prediction outcomes (the “warmer” the color, e.g., red, the more highly activated a particular region is). We performed CAM analyses on all 348 photographs in the validation and testing data set, and these CAM images were reviewed and interpreted by one of the authors (KL).

### Statistical analysis

To assess the performance of ONION, we calculated the AUC, sensitivity, specificity, and accuracy. The 95% confidence intervals (CIs) were estimated for all the performance metrics. Cohen’s kappa coefficients, AUC, sensitivity, and specificity were applied to compare the agreement scores among the results of the ONION system and ophthalmologists to the ground truth in the binary classification of ON or NAION. The Kappa result was interpreted as follows: values 0–0.2 as indicating no agreement, 0.21–0.39 as minimal, 0.40–0.59 as weak, 0.60–0.79 as moderate, 0.80–0.90 as strong, and >0.90 as almost perfect. Statistical analyses were carried out using SPSS Statistics version 23 (SPSS Inc., Chicago, IL, USA). The results of the ROCs obtained from the model and the ophthalmologists have merged accordingly into a figure using Adobe Illustrator CS6 version 24.0.1 (Adobe Inc., USA).

## Results

### Data set labels

The data set from 547 patients covered 871 eyes and 871 CFPs. The NAION group involved 159 patients (50.48%) in the bilateral affected eyes, while the ON group involved 164 patients (45.46%) in the bilateral affected eyes. The basic characteristics and the sample size of each data set are shown in Table 1.

**TABLE 1:** The basic characteristics of each patient group.

Group	NAION	ON	Total
Patients, *n*	315 (57.59)	232 (42.41)	547 (100)
Eyes, *n*	475 (54.54)	396 (45.46)	871 (100)
Age (mean ± SD)	53.67 ± 9.15 (31–83)	31.55 ± 12.08 (18–84)	
Female, *n* (%)	101 (32.06)	150 (64.66)	189 (45.89)
Bilateral affected, *n* (%)	159 (50.48)	164 (70.69)	323 (59.05)
Acute phase CFPs, *n* (%)	475 (54.54)	396 (45.46)	871 (100)
Disc edema, *n* (%)	396 (83.37)	117 (29.55)	561 (58.90)
Disc hemorrhages, *n* (%)	261 (54.95)	107 (27.02)	368 (42.25)
**ONION**			
Training data set, *n* (%)	285 (60.00)	238 (60.00)	523 (60.04)
Validation data set, *n* (%)	95 (20.00)	79 (20.00)	174 (19.98)
Testing data set, *n* (%)	95 (20.00)	79 (20.00)	174 (19.98)

ON, optic neuritis; NAION, non-arteritic anterior ischemic optic neuropathy; CFPs, color fundus photographs.

### Performance of the ONION system

In the validation data set, the ONION system distinguished between acute ON and NAION achieved the following mean performance: Time-consuming (23 s), AUC 0.903 (95% CI 0.827–0.947), accuracy 0.833 (95% CI 0.746–0.895), sensitivity 0.796 (95% CI 0.704–0.864), and specificity 0.865 (95% CI 0.783–0.920). Testing data set: time-consuming (17 s), AUC 0.902 (95% CI 0.832–0.944), accuracy 0.830 (95% CI 0.750–0.889), sensitivity 0.814 (95% CI 0.732–0.875), and specificity 0.841 (95% CI 0.762–0.897). The performance of the ONION system is shown in Table 2.

**TABLE 2:** Classification performance of the ONION system.

Data set	AUC (95% CI)	Sensitivity (95% CI)	Specificity (95% CI)	Accuracy (95% CI)
Validation data set	0.903 (0.827–0.947)	0.796 (0.704–0.864)	0.865 (0.783–0.920)	0.833 (0.746–0.895)
Testing data set	0.902 (0.832–0.944)	0.814 (0.732–0.875)	0.841 (0.762–0.897)	0.830 (0.750–0.88)

AUC, area under the receiver operating characteristic curve; CI, confidence interval.

### The ONION system had as good performance as a retinal expert

**FIGURE 1:**
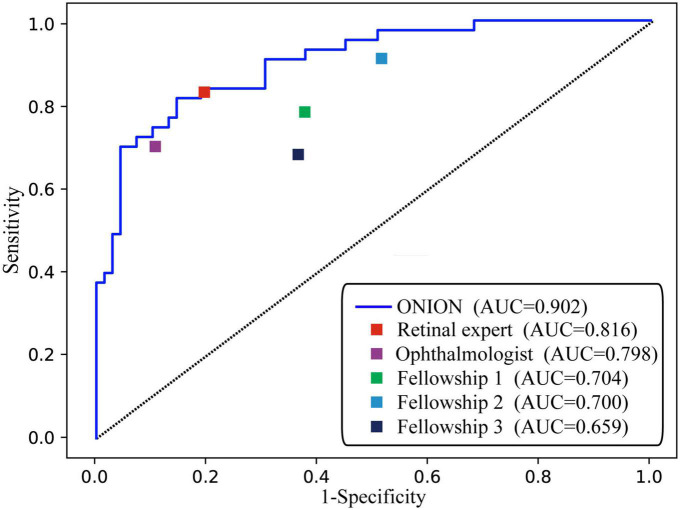
Performance of the ONION system and ophthalmologists. ROC of ONION system and five human graders for distinguishing acute ON from NAION. AUC, area under the receiver operating characteristic curve; NAION, non-arteritic anterior ischemic optic neuropathy; ON, optic neuritis.

As shown in Figure 1, the ONION system was comparable to that of the retinal expert [time-consuming (30 min), AUC: 0.816, sensitivity: 0.830, and specificity: 0.803] in the testing data set when distinguishing acute ON from NAION, which surpassed all the other ophthalmologists. Compared to the ground truth of the testing data set, Cohen’s kappa coefficients of ONION (κ = 0.805) exhibited slightly higher performance in disease identification than those of humans, even for the retinal expert (κ = 0.309–0.749) (Supplementary Table 1).

### Visualization of the ONION system

**FIGURE 2:**
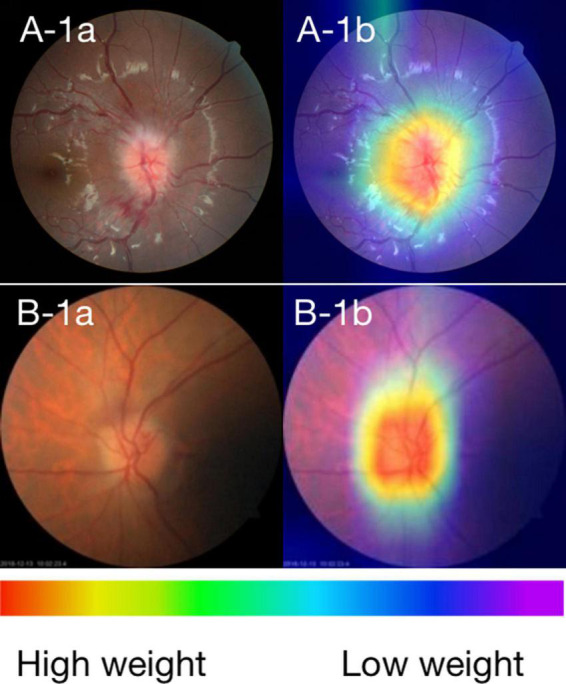
**(A)** Fundus photographs and **(B)** corresponding CAMs for ON and NAION. A-1a and A-1b: photographs of the ON 3 days after the onset. B-1a and B-1b: photographs of the NAION 1 week after the onset. The warmer the color, the higher its weight, and the more critical it is in a particular area.

In order to investigate the feature extraction location used by the ONION system during classification decisions, we performed CAM analysis on 348 images. As shown in Figure 2, typical CAM images with correct classification exhibited strong activation in the optic disc in 320 eyes (91.95%). Among these, 166 eyes (90.22%) were diagnosed with ON, while 154 eyes (93.90%) were diagnosed with NAION. By contrast, in typical CAM images with incorrect classification, we observed strong activation outside of the optic disc in 25 eyes (7.18%). Specifically, 18 eyes (9.78%) were diagnosed with ON and 10 eyes (6.09%) were diagnosed with NAION (Supplementary Figure 1 and Supplementary Table 2).

## Discussion

We attempted to distinguish between ON and NAION by AI based on CFPs alone from the acute phase, which achieved excellent performance with a mean AUC of 0.902. Its performance was comparable to that of a retinal expert and has surpassed that of other ophthalmologists. CAM revealed that the system produced a diagnosis using accurate distinguishing features, and the optic disc area was the most relevant to the diagnosis in the CFPs. These findings showed the encouraging application prospect of AI for differentiating these two common optic neuropathies in neuro-ophthalmology. In conditions where complex auxiliary examinations and neuro-ophthalmologist resources are not available, such as in underdeveloped areas, it is of unique advantage to make a reliable differentiation diagnosis based on CFPs alone.

This study contains the following advantages. Firstly, our study is the first to use AI to distinguish between ON and NAION during the acute phase. Secondly, our data is comprehensive and includes 6-month follow-up records for further diagnostic confirmation. In addition, our sample size is relatively large, which further ensures the accuracy of our model. Thirdly, we utilized the state-of-the-art EfficientNet algorithm to achieve expert-level performance on the CNN model and system implementation. The EfficientNet algorithm is widely regarded for achieving good classification accuracy through a model with moderate complexity, compared with other deep CNN algorithms. As demonstrated in the model evaluation results, our final output models achieved expert-level classification accuracy. Fourthly, we used CAM to further validate the accuracy of our AI model, which identified specific areas in the optic disc and to some extent demonstrated the interpretability of our AI model. Early diagnosis is an important issue of concern, especially for ophthalmic emergencies such as ON and NAION. While treatment may not always improve prognosis, early diagnosis and timely treatment can still enhance the patient’s quality of life.

Some attempts other than AI had been tried before to distinguish ON from NAION, such as B-scan ultrasonography (12), fluorescing angiography or laser speckle flowgraph (13), and MRI scanning (14). AI has been introduced into neuro-ophthalmology recently and has shown interesting results. AI could distinguish optic disc pallor from normal disc by machine learning and distinguish swollen discs of various optic neuropathies or pseudo-papilledema from normal discs using transfer learning based on CFPs alone (15, 16). Just recently, AI successfully distinguished papilledema caused by intracranial hypertension from other abnormalities and normal disc with an excellent AUC of 0.960 using a deep learning system based on CFPs alone (17, 18), and its performance was at least as good as two expert neuro-ophthalmologists (19). The same group further upgraded the system to distinguish mild/moderate/severe papilledema with an AUC of 0.930 (20). The differential diagnosis of ON and NAION is very challenging and essential in neuro-ophthalmology, but owing to the low incidence and lack of extensive and accurate training datasets, few studies that have used AI to distinguish between ON and NAION, and currently only Razaghi et al. have conducted research using optical coherence tomography scans as the basis for differentiating between these two conditions with the help of deep learning algorithm (11). In our study, we introduced AI into this field based on CFPs. A relatively extensive database and a reliable photograph label in our center enabled this ONION system to make an excellent performance for distinguishing ON from NAION in the acute phase. This was uncommon because even experienced ophthalmologists have difficulty identifying whether the cause of disc swelling is ON or NAION at the acute phase based on CFPs alone.

To evaluate the performance of the ONION system, we compared it to that of a retinal expert, an ophthalmologist, and three fellowships. The Cohen’s kappa coefficient indicates that retinal specialist possess significant reliability in distinguishing between ON and NAION. However, less experienced professionals may face difficulty in discerning between these two conditions, which is consistent with common sense. The result showed that our ONION system could be an effective and helpful tool for assisting ophthalmologists in distinguishing ON from NAION in the acute phase. So, we expect that in the future, a rapid differential diagnosis of NAION and ON could be made even in underdeveloped areas through AI and telemedicine technology.

Class activation map visualized the learning procedure of the ONION system, showing that the optic disc area contributed the most to system detection (91.95%), whether it is centered or partially centered. our use of AI, based purely on CFPs, demonstrated good performance in distinguishing ON from NAION. This result indirectly indicates that even though both ON and NAION can lead to optic disc edema, there may still be significant or subtle differences in the surface appearance of the optic disc.

Our study has some limitations: firstly, it was a single-center study. All of the participants in our study were from China. Therefore, there was no ethnic or racial diversity within our study population. While the lack of diversity in our sample limits the generalizability of our findings to other populations. Secondly, as it is a binary classification of the ON and NAION, it could not satisfy the real-world clinical requirements where other optic neuropathies are needed to differentiate. We need the next generation of the ONION system to identify other optic neuropathies, such as papilledema caused by intracranial hypertension and hereditary optic neuropathy. Thirdly, the ONION system was based on CFPs alone, a combination of CFPs with other modality images, such as VF, OCT, and MRI, might help get a more reliable and robust diagnostic ability. Fourthly, our study only compared the AI model with retinal specialists and general ophthalmologists, without comparison to neuro-ophthalmologists. However, we note that in some regions, the distribution of neuro-ophthalmologists is limited, making it difficult for many patients with ON and NAION to receive diagnosis from a neuro-ophthalmologist. Finally, AI interpretability is a topic that requires further exploration and attention in practical applications. We are committed to improving and enriching our model and seeking more interpretable and practical algorithms, so as to provide doctors with more accurate and reliable diagnostic and treatment recommendations in a wider range of medical environments.

## Conclusion

We developed a deep learning system ONION that could distinguish ON from NAION at the acute phases, with high sensitivity and specificity based on CFPs alone. Its efficiency was comparable to that of a retinal specialist. It required further prospective validation studies to prove its diagnostic ability in real-world clinical settings.

## Data availability statement

The raw data supporting the conclusions of this article will be made available by the authors, without undue reservation.

## Ethics statement

The studies involving human participants were reviewed and approved by the Zhongshan Ophthalmic Center. Written informed consent to participate in this study was provided by the participants’ legal guardian/next of kin. Written informed consent was obtained from the individual(s) for the publication of any potentially identifiable images or data included in this manuscript.

## Author contributions

HY and HL conceived and designed the study. SL and JH developed and validated the deep learning system. KL did the literature, designed the statistic analysis, and drafted the manuscript. HY finalized the diagnoses and labeled the fundus photographs. LW, XT, YF, XLiu, and XX participated in the data testing. KL and XLi collected the data. HY, WL, and JH critically revised the manuscript. All authors reviewed and approved the final version to be published.
