# Supplementary material for: Deep learning system for distinguishing optic neuritis from non-arteritic anterior ischemic optic neuropathy at acute phase based on fundus photographs
Source: Front Med (Lausanne). 2023 Jun 29;10:1188542. doi: 10.3389/fmed.2023.1188542 (PMC10339343; doi:10.3389/fmed.2023.1188542)
Supplement: Supplementary file 1 [file Data_Sheet_1.docx]

[SUPPLEMENTARY FIGURES 2](#_Toc137491722)

[Figure S1. Sample heatmaps where the features extracted by the CAMs were located outside the optic disc. 2](#_Toc137491723)

[SUPPLEMENTARY TABLES 3](#_Toc137491724)

[Table S1. The Performance (AUC, Sensitivity, Specificity, and Cohen’s Kappa) of the ONION system and ophthalmologists in the testing data set. 3](#_Toc137491725)

[Table S2. Statistical table of specific activation regions for Class Activation Mapping. 4](#_Toc137491726)

# SUPPLEMENTARY FIGURES

## Figure S1. Sample heatmaps where the features extracted by the CAMs were located outside the optic disc.


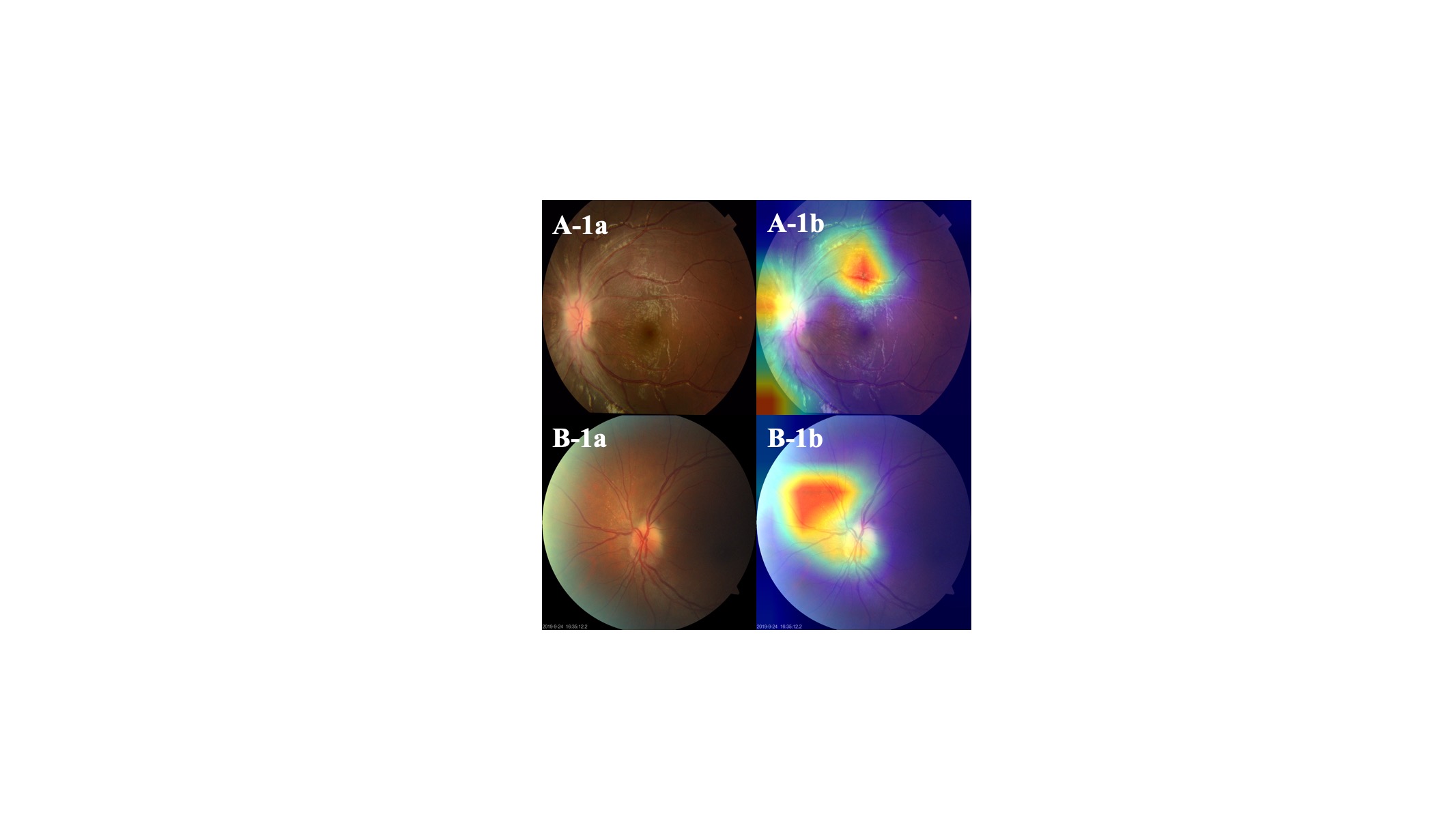


Fundus photographs(a) and corresponding CAMs(b) for ON(A) and NAION(B) . Abbreviations: CAM, Class activation map; NAION, Nonarteritic anterior ischemic optic neuropathy; ON, Optic neuritis.

# SUPPLEMENTARY TABLES

## Table S1. The Performance (AUC, Sensitivity, Specificity, and Cohen’s Kappa) of the ONION system and ophthalmologists in the testing data set.

| Test dataset | AUC | Sensitivity | Specificity | Kappa | Time-consuming |
| --- | --- | --- | --- | --- | --- |
| **Acute phase** |  |  |  |  |  |
| ONION | 0.902 | 0.814 | 0.841 | 0.805 | 17 seconds |
| Retinal expert | 0.816 | 0.830 | 0.803 | 0.749 | 30 minutes |
| Ophthalmologist | 0.798 | 0.702 | 0.894 | 0.609 | 1 hours |
| Fellowship 1 | 0.704 | 0.787 | 0.621 | 0.391 | 2.5 hours |
| Fellowship 2 | 0.700 | 0.915 | 0.485 | 0.366 | 2 hours 15 minutes |
| Fellowship 3 | 0.659 | 0.681 | 0.636 | 0.309 | 2 hours |

Abbreviations: AUC, Area under the receiver operating characteristic curve.

## Table S2. Statistical table of specific activation regions for Class Activation Mapping.

| Labels/Regions | Optic disc | Non-optic disc | Total |
| --- | --- | --- | --- |
| ON, n (%) | 166 (90.22) | 18 (9.78) | 184 (52.87) |
| NAION, n (%) | 154 (93.90) | 10 (6.09) | 164 (47.13) |
| Total, n (%) | 320 (91.95) | 25 (7.18) | 348 (100) |

Abbreviations: ON, Optic neuritis; NAION, Non-arteritic anterior ischemic optic neuropathy.
